# The pharmacokinetics of high-dose methotrexate in people living with HIV on antiretroviral therapy

**DOI:** 10.1007/s00280-015-2940-3

**Published:** 2015-12-22

**Authors:** Alessia Dalla Pria, Maggie Bendle, Ramya Ramaswami, Marta Boffito, Mark Bower

**Affiliations:** Department of Oncology, Chelsea and Westminster Hospital, 369 Fulham Road, London, SW10 9NH UK; Department of HIV Medicine, Chelsea and Westminster Hospital, London, SW10 9NH UK

**Keywords:** Chemotherapy, HIV, Methotrexate, Pharmacokinetics

## Abstract

**Purpose:**

Clinical outcomes for lymphoma in people living with HIV (PLWH) are similar to those in the general public. However, a number of concerns remain including pharmacological interactions between cytotoxic chemotherapy and antiretroviral therapy (ARVs). Much attention has focussed on pharmacokinetic interactions attributable to effects on hepatic microsomal enzymes, but not on competition for the renal organic anion transport system. High-dose (3 g/m^2^) intravenous methotrexate (MTX) is part a of (R)-CODOX-M/IVAC chemotherapy regimen for HIV-associated Burkitt/Burkitt-like lymphoma (BL/BLL). We investigated MTX pharmacokinetics and evaluated the effects of renal function (eGFR), age and use of different classes of ARVs.

**Methods:**

Forty-three PLWH treated with ARVs and (R)-CODOX-M/IVAC are included in the analysis. Plasma MTX concentration was measured (ARK TM MTX assay, VITROS^®^ 5600) daily after administration until levels were <0.04/mmol/L. MTX elimination half-life was correlated with age, renal function and antiretroviral regimen.

**Results:**

One hundred and fifty timed plasma MTX levels were collected. The median MTX elimination half-life was 21.7 h (range 9.4–204.4). MTX elimination half-life was not influenced by age (*p* = 0.71), eGFR (*p* = 0.67) or use of non-nucleoside reverse transcriptase inhibitors (NNRTIs) or integrase inhibitors (*p* = 0.15). Similarly, different NRTI backbones did not affect MTX elimination kinetics (*p* = 0.68), despite the potential overlapping competition for active renal tubular transporters between MTX and tenofovir.

**Conclusion:**

Although there is potential competition for active renal tubular transporters between MTX and tenofovir, no prolongation of MTX half-life was observed. These findings are reassuring to clinicians managing patients with dual diagnoses.

## Introduction

Human immunodeficiency virus (HIV) infection substantially increases the risk of developing non-Hodgkin lymphoma (NHL) [1]. This is attributable to impaired cellular immunity and increased susceptibility to oncogenic viruses. The two most frequent histological subtypes of NHL subtypes in people living with HIV (PLWH) are diffuse large B cell lymphoma (DLBCL) and Burkitt lymphoma (BL) which are AIDS-defining illnesses. Burkitt/Burkitt-like lymphoma (BL/BLL) accounts for 25–40 % of HIV-associated NHL [2]. In the HIV-negative population, BL is a highly curable cancer if treated with intensive, short duration, chemotherapy regimens. High intravenous doses of the anti-metabolite methotrexate (MTX), with folinic acid rescue, are an integral part of the treatment.

(R)-CODOX-M/IVAC (rituximab, cyclophosphamide, vincristine, doxorubicin, methotrexate/ifosfamide, etoposide, cytarabine) is the standard chemotherapy regimen for BL/BLL in the UK and includes 3 g/m^2^ methotrexate. This regimen is used with antiretroviral therapy in PLWH diagnosed with BL/BLL and has been shown to achieve outcomes comparable to the HIV-negative patients (complete remission rate of 84 % and a 2-year overall survival of 73 %) [3–6].

However, chemotherapy in PLWH may be complicated by clinically significant pharmacokinetic interactions between cytotoxic drugs and antiretrovirals (ARVs). No data on the pharmacokinetics (PK) of high-dose MTX in PLWH are available. We investigated MTX PK and evaluated the effects of renal function (eGFR), age and use of different classes of ARVs.

## Methods

All people living with HIV (PLWH) treated with (R)-CODOX-M/IVAC for HIV-associated Burkitt/Burkitt-like lymphoma between 2007 and 2014 at the National Centre for HIV Malignancy, Chelsea and Westminster Hospital, London, are included in the analysis. As part of routine care, following methotrexate infusion (3 g/m^2^) plasma was collected and sent to Great Ormond Street Hospital for Children in London for MTX concentration measurement (ARK TM MTX assay, VITROS 5600) daily. Treatment with folinic acid rescue and urinary alkalinization continued until MTX concentrations <0.05 mmol/L were achieved. No patients received glucarpidase as salvage for methotrexate toxicity. Clinical data on renal, liver function and ARV regimen (nucleoside reverse transcriptase inhibitors (NRTIs) backbones and use of third agent) were collected. Comparison of MTX elimination half-life (t½) with relevant clinical variables was performed using *t* test (StatView 1989).

## Results

We treated 43 PLWH (8 women, 35 men) with HIV-associated BL/BLL with (R)-CODOX-M/IVAC. At the start of chemotherapy, the mean age was 42 years (range 24–71) and the mean duration of HIV infection was 28 months (range 0–295). Two (5 %) had a prior AIDS-defining illness, and 12 (28 %) were established on combination antiretroviral therapy (cART) prior to lymphoma diagnosis, of whom ten (83 %) had an undetectable plasma HIV viral load. For the whole cohort, the median CD4 cell count was 225 cells/mm^3^ (range 10–864), the median CD4 percentage 13 % (range 2–39), the median CD8 cell count 884 cells/mm^3^ (range 272–2065) and the median CD8 percentage 55 % (range 22–77). The median plasma HIV viral load for the cohort was 10,860 copies/mL (range 0–1.9 M).

The combination antiretroviral therapy that was concomitantly prescribed with the methotrexate included 36 (84 %) patients on a nucleotide/side reverse transcriptase inhibitor (NRTI) backbone of tenofovir/emtricitabine backbone and seven (16 %) on a backbone of abacavir/lamivudine. Twenty-two (51 %) patients were on integrase inhibitors (INI), 18 (42 %) on non-NRTIs (NNRTIs), one (2 %) on a ritonavir-boosted protease inhibitor, one (2 %) on both NNRTI and INI, and one (2 %) on INI and maraviroc.

**Fig. 1:**
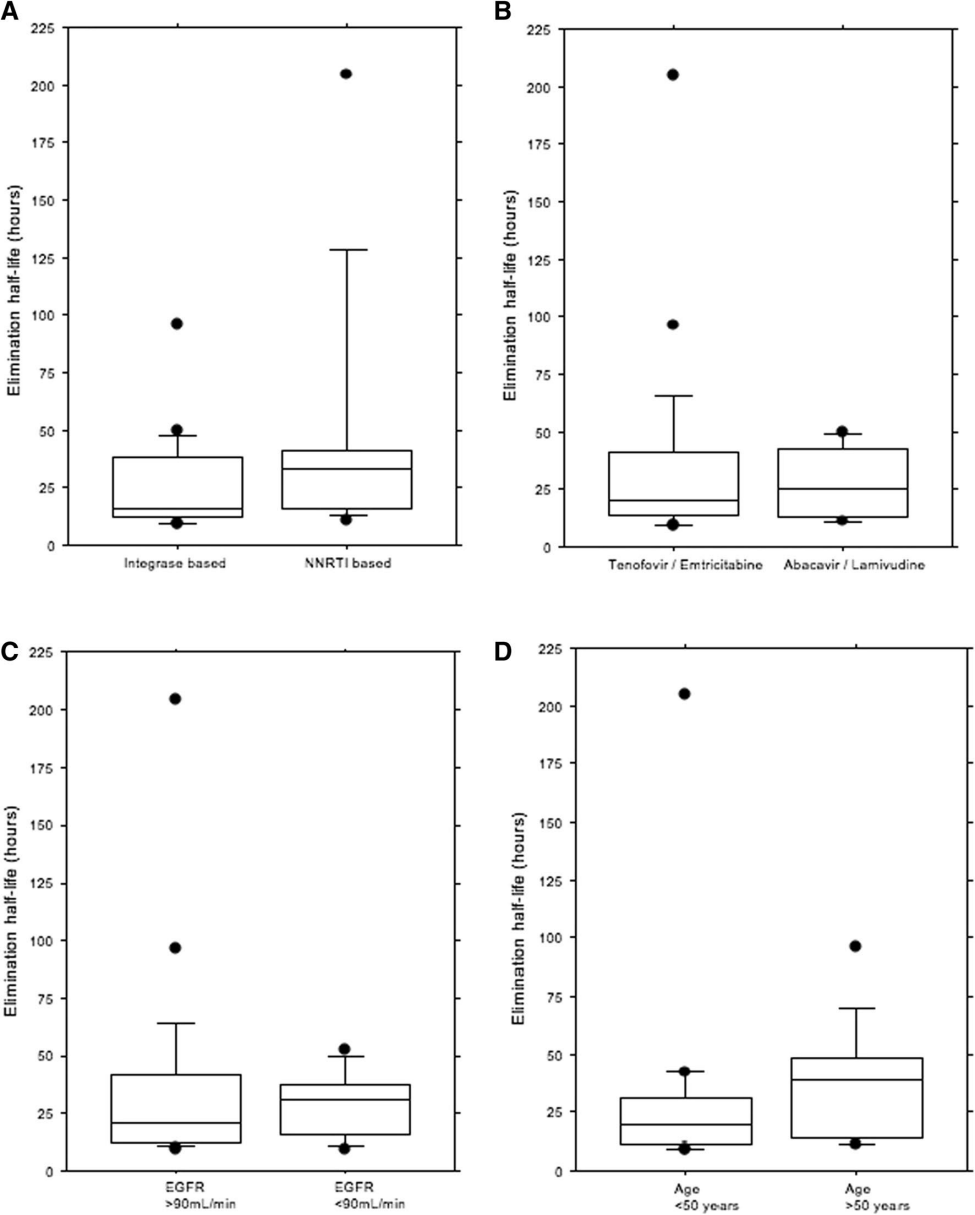
Half-life elimination of MTX (*n* = 29 cycles). **a** Integrase-based versus NNRTI-based ARV group (*p* = 0.15). **b** TDF/FTX versus ABC/3TC ARV backbone (*p* = 0.68). **c** Elimination of MTX was not affected by eGFR (*p* = 0.67). **d** Elimination of MTX was not affected by age (*p* = 0.75)

A total of 183 methotrexate levels were assayed following administration of 3 g/m^2^ methotrexate, but accurate timings of the sample collection were not available for 33 samples. The 150 timed MTX levels from 43 PLWH included 18 samples from women and 132 from men. The median half-life (t½) of methotrexate was 21.7 h (range 9.4–204.4). The elimination half-life of methotrexate was not affected by eGFR (*p* = 0.67) (Fig. 1c) or age (*p* = 0.71) (Fig. 1d). There was no difference in methotrexate elimination kinetics between patients on non-nucleoside reverse transcriptase inhibitors (NNRTIs) and those on integrase inhibitors (*p* = 0.15) (Fig. 1a). There was no difference in methotrexate elimination kinetics between patients on different NRTI backbones (*p* = 0.68) (Fig. 1b).

Elevated serum concentrations of methotrexate at 24, 48 and 72 h (in excess of 20, 2 and 0.2 µM, respectively) were recorded for 31/150 (21 %) time points. An elevated level was not associated with use of NNRTIs rather than integrase inhibitors (*p* = 0.8) or by NRTI backbones (*p* = 0.9). Following methotrexate, only one patient developed CTCv4.0 grade 1 acute nephrotoxicity, and his elimination half-life of methotrexate was 38.8 h.

## Discussion

Pharmacokinetic drug interactions can significantly affect the efficacy and toxicity of chemotherapy. The most frequent mechanism of these interactions is the inhibition and induction of hepatic cytochrome P450 (CYP) microsomal enzymes by co-administered drugs. The CYP enzymes are responsible for the vast majority of drug metabolic deactivation and clearance. Many drugs including antiretroviral agents increase or decrease the activity of various CYP isoenzymes by inducing their biosynthesis or by inhibiting their activity, resulting in adverse drug interactions. For example, ritonavir is a potent inhibitor of CYP3A4 activity, leading to increased bioavailability of many of the substrates of CYP3A4 including cytotoxic chemotherapy agents, and resulting in overdosing and excess toxicity. This pharmacological drug interaction has been shown to be of clinical significance in treating patients with HIV-associated lymphoma. Patients receiving ritonavir-boosted PI-based cART experienced greater myelosuppression and subsequently increased the risk of hospitalization for severe neutropenic sepsis with CDE (cyclophosphamide, doxorubicin and etoposide) for HIV-associated NHL [7]. Since cyclophosphamide, vincristine, doxorubicin, ifosfamide and etoposide, all components of CODOX-M/IVAC chemotherapy, are substrates of CYP3A4, ritonavir-boosted PIs are avoided and all but one of our patients were prescribed NNRTI-based and integrase inhibitor-based ARV regimens. Furthermore, ritonavir has been shown to inhibit the activity of organic anion transporters (OATs) and organic cation transporters (OCTs), which are expressed in numerous organs, including the kidneys and the liver, and hence may be responsible for additional drug interactions at the cellular level [8].

Methotrexate is eliminated by renal clearance with almost 90 % excreted unchanged in the urine. Although the mechanism of MTX excretion in the human kidney has not been completely elucidated, the finding that MTX clearance exceeds creatinine clearance in several studies suggests that there is active tubular secretion as well as glomerular filtration [9]. The tubular secretion of methotrexate is mediated by an organic anion transport system, and recent molecular studies have identified multiple candidates for organic anion transporters that can mediate methotrexate transport in the kidney, including basolateral OAT1 and OAT2, multidrug resistance-associated protein 1(MRP1) and MRP2, and the kidney-specific organic anion transporters OATK1 and OATK2. Efficient OAT-mediated basolateral uptake could result in high intracellular drug concentrations, whereas at the apical-side MRPs control the intracellular concentration by active drug efflux into urine [10]. The pharmacokinetics of MTX is thus prone to drug–drug interactions that occur at the renal transporter level. Many drugs inhibit renal excretion of MTX and may potentially increase treatment-related toxicity, for example NSAIDs, phenytoin, ciprofloxacin, penicillin drugs, probenecid, amiodarone and proton pump inhibitors [11]. Because urinary excretion is the main route of elimination, MTX can reach high concentration in the renal tubules causing nephrotoxicity [12, 13].

There is a clear relationship between plasma MTX levels, adverse events and treatment efficacy with important individual differences [14]. This individual variability can be linked to germline polymorphisms of genes involved in drug absorption, metabolism, excretion, cellular transport, and/or effector targets and pathways [15]. Nevertheless, the incidence of toxicity can be substantially reduced by a combination of urine alkalinization and optimal hydration to encourage renal clearance, folinic acid rescue to negate the effects of MTX on normal cells and plasma MTX drug monitoring to determine when it is safe to cease these measures [12]. All this procedures are carefully applied in the CODOX-M/IVAC protocol for our HIV-positive patients with BL/BLL lymphoma at Chelsea and Westminster Hospital.

In this study, we evaluated the clearance of MTX in PLWH receiving 3 g/m^2^ MTX as part of the CODOX-M/IVAC regimen with concomitant cART. While the definition of high-dose MTX is not clear in the literature, the overall plasma half-life (t½) of MTX following intravenous administered as a dose >30 mg/m^2^ is 8–12 h, although this may be longer when higher doses such as 3 g/m^2^ are administered [11].

The median plasma MTX half-life in our study population was 21.7 h (range 9.4–204.4). There was no significant difference in MTX clearance in patients with reduced eGFR (p = 0.67); this is surprising since impaired renal function reduces MTX elimination, but only 29 (19 %) samples were taken from patients with estimated GFR below 90 mL/min. Similarly, age did not influence plasma MTX half-life (*p* = 0.71). This has been previously demonstrated in the HIV-negative setting where no significant reduction in MTX clearance was observed in older (>60 years old) patients treated with 4 g/m^2^ for primary central nervous system lymphoma [16].

The influence of co-administered ARVs on MTX elimination was evaluated by class of antiretroviral. There was no statistically significant difference in half-life between patients on non-nucleoside reverse transcriptase inhibitors (NNRTIs) and those on integrase inhibitors (*p* = 0.15). Tenofovir disoproxil fumarate (TDF) is recommended as first-line nucleotide reverse transcriptase inhibitor (NRTI) for HIV-1 treatment. Prolonged treatment with TDF has been linked to nephrotoxicity characterized by eGFR decline and proximal tubular dysfunction, especially if used in combination with boosted PIs [17]. TDF is a pro-drug that is converted to tenofovir and subsequently cleared by renal glomerular filtration and active proximal tubular excretion. The nephrotoxicity is related to intracellular accumulation of tenofovir in proximal tubule cells that results in mitochondrial DNA depletion and cytotoxicity. Tenofovir renal proximal tubular toxicity is regulated by OAT1 (drug influx at basolateral side of tubular cells) and MRP4 (drug efflux in the pre-urine) transporters [18]. Thus, there is a clear potential for competition for active renal basolateral organic anion transporter 1 when tenofovir and methotrexate are co-administered, which could lead to decreased clearance and increased exposure to either drug and therefore to a drug interaction at this level even in the absence of ritonavir-boosted protease inhibitors. Reassuringly, in our cohort of patients, there was no difference in MTX half-life between patients receiving a TDF-based backbone compared to those on TDF-sparing backbones (*p* = 0.68).

This study has some limitations. Firstly, data have been retrospectively collected, and MTX concentration testing sent in a real-life clinical practice and not in a pre-scheduled clinical trial setting. Important genetic polymorphisms influence MTX metabolism, causing pharmacokinetic variability, but no pharmacogenetic analysis was undertaken in this cohort. Finally, the influence of other medications such as NSAIDs, penicillin drugs and proton pump inhibitors has not been evaluated.

## Conclusion

Pharmacological drug–drug interactions in the management of HIV-associated lymphomas have had important clinically significant effects. Plasma methotrexate levels influence both its efficacy and its toxicity, and the elimination is by renal clearance. In an analysis of 43 people living with HIV who were treated with 3 g/m^2^ MTX as part of chemotherapy for BL/BLL, the elimination half-life of MTX was not affected by eGFR, age or ARV regimen. This is despite the potential overlapping competition for active renal tubular transporters between MTX and tenofovir. These findings are reassuring to clinicians managing patients with dual diagnoses, but prospective data on the PK and the pharmacodynamics of ARV and cytotoxics are warranted to improve treatment of cancer in PLWH.

## References

[1] Cote TR, Biggar RJ, Rosenberg PS, Devesa SS, Percy C, Yellin FJ, Lemp G, Hardy C, Geodert JJ, Blattner WA (1997). Non-Hodgkin’s lymphoma among people with AIDS: incidence, presentation and public health burden. AIDS/Cancer Study Group. Int J Cancer.

[2] Biggar RJ, Chaturvedi AK, Goedert JJ, Engels EA (2007). AIDS-related cancer and severity of immunosuppression in persons with AIDS. J Natl Cancer Inst.

[3] Mead GM, Barrans SL, Qian W, Walewski J, Radford JA, Wolf M, Clawson SM, Stenning SP, Yule CL, Jack AS (2008). A prospective clinicopathologic study of dose-modified CODOX-M/IVAC in patients with sporadic Burkitt lymphoma defined using cytogenetic and immunophenotypic criteria (MRC/NCRI LY10 trial). Blood.

[4] Oriol A, Ribera JM, Bergua J, Gimenez Mesa E, Grande C, Esteve J, Brunet S, Moreno MJ, Escoda L, Hernandez-Rivas JM, Hoelzer D (2008). High-dose chemotherapy and immunotherapy in adult Burkitt lymphoma: comparison of results in human immunodeficiency virus-infected and noninfected patients. Cancer.

[5] Barnes JA, Lacasce AS, Feng Y, Toomey CE, Neuberg D, Michaelson JS, Hochberg EP, Abramson JS (2011). Evaluation of the addition of rituximab to CODOX-M/IVAC for Burkitt’s lymphoma: a retrospective analysis. Ann Oncol.

[6] Alwan F, He A, Montoto S, Kassam S, Mee M, Burns F, Edwards S, Wilson A, Tenant-Flowers M, Marcus R, Ardeshna KM, Bower M, Cwynarski K (2015). Adding rituximab to CODOX-M/IVAC chemotherapy in the treatment of HIV-associated Burkitt lymphoma is safe when used with concurrent combination antiretroviral therapy. AIDS.

[7] Bower M, McCall-Peat N, Ryan N, Davies L, Young AM, Gupta S, Nelson M, Gazzard B, Stebbing J (2004). Protease inhibitors potentiate chemotherapy-induced neutropenia. Blood.

[8] Griffin L, Annaert P, Brouwer KL (2011). Influence of drug transport proteins on the pharmacokinetics and drug interactions of HIV protease inhibitors. J Pharm Sci.

[9] Monjanel S, Rigault JP, Cano JP, Carcassonne Y, Favre R (1979). High-dose methotrexate: preliminary evaluation of a pharmacokinetic approach. Cancer Chemother Pharmacol.

[10] Takeuchi A, Masuda S, Saito H, Doi T, Inui K (2001). Role of kidney-specific organic anion transporters in the urinary excretion of methotrexate. Kidney Int.

[11] Green MR, Chowdhary S, Lombardi KM, Chalmers LM, Chamberlain M (2006). Clinical utility and pharmacology of high-dose methotrexate in the treatment of primary CNS lymphoma. Expert Rev Neurother.

[12] Twelves CJ (1986). Folinic acid rescue and methotrexate toxicity. Lancet.

[13] El-Badawi MG, Abdalla MA, Bahakim HM, Fadel RA (1996). Nephrotoxicity of low-dose methotrexate in guinea pigs: an ultrastructural study. Nephron.

[14] Zhang W, Zhang Q, Tian X, Zhao H, Lu W, Zhen J, Niu X (2015). Population pharmacokinetics of high-dose methotrexate after intravenous administration in Chinese osteosarcoma patients from a single institution. Chin Med J (Engl).

[15] Radtke S, Zolk O, Renner B, Paulides M, Zimmermann M, Moricke A, Stanulla M, Schrappe M, Langer T (2013). Germline genetic variations in methotrexate candidate genes are associated with pharmacokinetics, toxicity, and outcome in childhood acute lymphoblastic leukemia. Blood.

[16] Jahnke K, Korfel A, Martus P, Weller M, Herrlinger U, Schmittel A, Fischer L, Thiel E (2005). High-dose methotrexate toxicity in elderly patients with primary central nervous system lymphoma. Ann Oncol.

[17] Ryom L, Mocroft A, Kirk O, Worm SW, Kamara DA, Reiss P, Ross M, Fux CA, Morlat P, Moranne O, Smith C, Lundgren JD (2013). Association between antiretroviral exposure and renal impairment among HIV-positive persons with normal baseline renal function: the D:A: D study. J Infect Dis.

[18] Kohler JJ, Hosseini SH, Green E, Abuin A, Ludaway T, Russ R, Santoianni R, Lewis W (2011). Tenofovir renal proximal tubular toxicity is regulated by OAT1 and MRP4 transporters. Lab Invest.

